# Differential expression of store-operated calcium- and proliferation-related genes in hepatocellular carcinoma cells following TRPC1 ion channel silencing

**DOI:** 10.1007/s11010-016-2776-0

**Published:** 2016-07-22

**Authors:** Cigdem Selli, Dominic A. Pearce, Andrew H. Sims, Metiner Tosun

**Affiliations:** 1 Department of Pharmacology, Faculty of Pharmacy, Ege University, 35040 Izmir, Turkey; 2 Applied Bioinformatics of Cancer, Institute of Genetics and Molecular Medicine, Edinburgh Cancer Research Centre, Crewe Road South, Edinburgh, EH4 2XU UK; 3 Faculty of Medicine, Izmir University of Economics, 35330 Izmir, Turkey

**Keywords:** Huh7, Hepatocellular carcinoma, Calcium, Proliferation, Microarray

## Abstract

TRPC1 and store-operated Ca^2+^ (SOC) entry have previously been associated with hepatocellular carcinoma cell proliferation. The aim of the study was to determine genes and processes associated with *TRPC1* down-regulation and the resulting increase of SOC entry and decrease in hepatocellular carcinoma cell proliferation. For this purpose, transcriptome analysis was performed to determine differentially expressed genes in *TRPC1*-silenced Huh7 cells. SOC entry- and proliferation-related genes correlated with *TRPC1* down-regulation were also examined. Changes in SOC entry and cell proliferation were monitored in the *TRPC1*-silenced and parental cells and found to be significantly increased and decreased, respectively, in *TRPC1*-silenced cells. A total of 71 genes were significantly differentially expressed (40 up- and 31 down-regulated), including four mitogen-activated protein kinase (MAPK) signalling-associated genes. STIM1 levels were significantly up-regulated and negatively correlated with TRPC1 levels. In addition, expression of two cell cycle regulation genes, CDK11A/11B and URGCP, was observed to decrease, whereas ERBB3 and FGFR4, pro-survival genes, increased significantly in *TRPC1*-silenced cells. In conclusion, these results suggest reciprocal alterations in TRPC1 and STIM1 levels and a role for STIM1 in the regulation of SOC entry in *TRPC1*-silenced Huh7 cells. In addition to TRPC1, STIM1 may participate in Huh7 cell proliferation by regulating SOC entry. Alterations in MAPK signalling genes may be involved in diminished cell proliferation in *TRPC1*-silenced Huh7 cells. Similarly, changes in cell cycle regulating genes in *TRPC1*-silenced cells indicate possible cell cycle arrest along with compensatory up-regulation of ERBB3 growth factor receptor—amongst others—to maintain hepatocellular carcinoma cell proliferation.

## Introduction

Hepatocellular carcinoma is the most common type of primary liver cancer and the third leading cause of cancer-related deaths worldwide, and is associated with poor prognosis, few effective therapeutic options and resistance to both chemotherapy and radiotherapy. The Wnt/β-catenin pathway appears to be commonly deregulated in hepatocellular carcinogenesis [1]. Mitogen-activated cascades are also critical, where inhibition of mitogen-activated protein kinase (MAPK) and the phosphatidylinositide 3-kinase/Akt/mammalian target of rapamycin PI3K/Akt/mTOR pathways have been shown to inhibit tumour growth [2]. A multi-target kinase inhibitor, sorafenib, that inhibits tumour angiogenesis and proliferation is currently clinically available for the treatment of hepatocellular carcinoma [3], although partial and complete response rates remain relatively low, 30.8 and 2.6 %, respectively [4]. However, despite sorafenib’s success, effective treatment options are limited, needing further investigation to identify disrupted signalling pathways that may eventually lead to uncontrolled proliferation of hepatocellular carcinoma cells. Transient receptor potential canonical 1 (TRPC1) down-regulation has previously been shown to be associated with hepatocellular carcinoma cell proliferation [5], and therefore TRPC1 may exist as a potential novel drug target.

TRP channels are a subfamily of the TRP ion channel superfamily, first discovered in *Drosophila melanogaster* composed of approximately 30 members identified in mammalian cells [6]. The superfamily has been classified into seven subfamilies: TRPC (canonical), TRPM (melastatin), TRPV (vanilloid), TRPA (ankyrin), TRPP (polycystin), TRPML (mucolipin), and TRPN (NOMPC, no mechanoreceptor potential C). TRPC1 has been suggested to be an essential component of store-operated Ca^2+^ (SOC) entry channel by forming a multimeric complex with other TRPCs [7]. SOC entry, activated in response to endoplasmic reticulum (ER) Ca^2+^ depletion and suggested to be an ER Ca^2+^ maintenance mechanism, controls a diverse catalogue of cellular functions, including cell cycle regulation [8].

The mechanism of activation for SOC entry is still unclear. STIM1, a calcium sensor located in the ER membrane, has been suggested to link depletion of intracellular Ca^2+^ stores and SOC entry through Orai1 channels [9]. STIM1 and Orai1-mediated SOC entry is apparent in vascular smooth muscle proliferation [10], but also plays a role in hepatocellular carcinoma cell migration and invasion [11]. The interaction between STIM1, Orai1, and TRPC proteins in mediating SOC entry and their mutual regulation remains controversial and requires further investigation. STIM1 was shown to bind TRPC1 and to be essential for activation and gating TRPC channels [12, 13]. Conversely, TRPC and STIM1/Orai1 signalling have also been suggested to occur independently in distinct plasma membrane domains [14].

We have previously observed SOC entry to increase following *TRPC1* silencing, suggesting a negative regulatory role of TRPC1 in SOC entry both in vascular smooth muscle and hepatocellular carcinoma cells [15, 16]. The potential negative regulatory role of TRPC1 in SOC entry has recently been reviewed by Dietrich et al. [17]. TRPC6 has also been reported to be up-regulated following *TRPC1* silencing in vascular smooth muscle cells [15]. In addition, Huh7 cell proliferation has been shown to be linked with TRPC6 and SOC entry, but not with TRPC1 [5]. This contradicts our own observation that Huh7 cell proliferation is suppressed in *TRPC1*-silenced cells without any alteration to TRPC6 levels [16].

We suggest that these discrepancies may arise from different compositions of SOC entry channels and regulatory mechanisms. Therefore, this study seeks to determine which genes and pathways become deregulated upon *TRPC1* silencing, to better understand the role of TRPC1 in SOC entry and hepatocellular carcinoma cell proliferation. For this purpose, whole-transcriptome gene expression profiling was performed in *TRPC1*-silenced Huh7 hepatocellular carcinoma cells. We, for the first time, describe candidate genes related to SOC entry up-regulation and suppression of proliferation following *TRPC1* silencing.

## Materials and methods

### Cell culture

The well-differentiated human hepatocellular carcinoma cell line, Huh7 [18], was cultured in DMEM (Biological Industries) supplemented with 10 % foetal bovine serum (FBS, Gibco), 2 mM l-glutamine (Gibco), and 0.1 mM non-essential aminoacid solution (Gibco). Huh7 cells, originally from Jack Wands Laboratory at Massachusetts General Hospital, Boston, MA, were a gift provided by Mehmet Ozturk, DEU University, Turkey. Cells were tested for authenticity in 2010 and were also regularly checked for mycoplasma contamination using MycoAlert Mycoplasma Detection kit (Lonza) in our laboratory.

### TRPC1 gene silencing

The silencing sequence (5′-GAACAUAAAUUGCGUAGAU-3′) targeting 361st–379th nucleotides of TRPC1 mRNA (NM_003304) was cloned into a pSUPERIOR.retro.neo+gfp vector (Oligoengine). Cells were transfected with 2 µg silencing vector and an empty vector as a negative control, using 6 µl FugeneHD transfection reagent (Roche Applied Science). Transfection efficiency was determined by monitoring the GFP signal using fluorescence microscopy (IX71, Olympus), and cells with efficiency greater than 70 % were used in further experiments.

### Microarray experiments

Total RNA was isolated from TRPC1 silencing vector (siTRPC1) and empty vector-transfected (control) cells following 48-h incubation using the instructions provided in the High Pure RNA Isolation Kit (Roche Applied Science). The incubation time was chosen based on our previous report [16]. 500 ng total RNA was amplified and biotin labelled using the Illumina Total Prep RNA Amplification Kit (Ambion). Biotinylated cRNA (750 ng) was hybridised at 58 °C for 16 h to HumanHT-12 v3 expression BeadChip (Direct Hybridization Assay Kit, Illumina). The BeadChip was washed, blocked, and scanned using (Illumina BeadArray Reader), and Cy3 signal intensity was measured.

Data quality was assessed using GenomeStudio, all system control values were within the expected ranges. Background fluorescence representing signals from non-specific dye binding and/or cross-hybridization were subtracted from all other probe intensities using GenomeStudio. R and BioConductor packages were used for analysis. Following quantile normalisation using lumi, Rank Product analysis [19] was performed using the RankProd package to determine differentially expressed genes. Pathway analysis was performed using DAVID Bioinformatics Resources 6.7 (functional annotation clustering) [20]. Raw and processed microarray data have been submitted to GEO database (GSE77386).

### Quantitative real-time RT-PCR and western blotting

A detailed protocol was described formerly [16]. We previously showed that following silencing vector transfection, TRPC1 mRNA levels were reversibly inhibited at 24 h, a significant decrease at 48 h (*P* ≤ 0.05, *n* = 4–5), and recovered at 72 h with significant inhibition in protein levels at 72 h [16]. Based on this, following 48-h vector incubation, RNA was isolated using High Pure RNA Isolation Kit (Roche Applied Science). PCR experiments were performed using FastStart DNA Master SYBR Green I kit and LightCycler 1.5 (Roche Applied Science). TRPC1 mRNA levels normalised to that of internal 18S rRNA ([TRPC1]/[18S rRNA] × 1000). The effects of TRPC1 silencing on TRPC1 protein levels were measured by western blot following 72-h vector incubation as described previously [16]. Briefly, following separation on 8 % SDS-PAGE, proteins were transferred to a nitrocellulose membrane using a dry blotting system (iBlot, Invitrogen). Protein levels were normalised to that of internal β-actin and represented as relative optical density ([TRPC1]/[β-actin] × 1000).

### Intracellular calcium measurements

Huh7 cells were grown on 96-well microplates and imaging performed 72 h following silencing vector transfection. This incubation time was chosen based on our previous report showing significant changes in TRPC1 protein levels and SOC entry following 72-h vector incubation [16]. Cells were incubated for 30 min at room temperature in HEPES-buffered saline in mM: NaCl 135, KCl 5.9, MgCl_2_ 1.2, CaCl_2_ 1.5, HEPES 11.6, NaHCO 3 5, glucose 11.5, at pH 7.3 with 1 mg/ml BSA and 2.5 mM fura-2/AM (Molecular Probes). Cells were then rinsed twice via 15-min incubations. Changes in intracellular Ca^2+^ levels were monitored using a microplate reader (Victor3, Perkin Elmer) and expressed as a ratio based on fluorescence emissions at 510 nm, and monitored sequentially upon excitations at 340 and 380 nm (340/380). The results indicate the average Ca^2+^ levels in a group of cells, and Ca^2+^ levels in subpopulations such as transfected and/or viable cells could not be discriminated in this system. Cyclopiazonic acid (CPA) at 10 µM concentration that depletes ER-stored Ca^2+^ was used to activate SOC entry. Cells were exposed to the Ca^2+^-free solution, then CPA was applied and, following a transient increase in Ca^2+^ levels due to store depletion, Ca^2+^ was added to initiate SOC entry. Peak responses to agents and applications were evaluated due to time-dependent decays in plateau responses.

### Doubling time

Huh7 cells were transfected in 6-well plates and after 72-h incubation, 1250 cells/well were seeded into E-plate 96. Cell proliferation was monitored every 30 min for 96 h using xCELLigence system (Roche Applied Science) that allows performing real-time and label-free cellular analyses. Changes in proliferation were expressed as cell index which is defined as (*R*_n_−*R*_b_)/15, where *R*_b_ is the background impedance and *R*_n_ is the impedance of the well with cells. Doubling time was determined with RTCA software 1.2.1 (Roche Applied Science). The time required to reach a cell index value of 4 from 2 was calculated for each group.

### BrDU incorporation assay

Cell proliferation was also evaluated by measuring 5-bromo-2′-deoxyuridine (BrDU) incorporation during DNA synthesis using colorimetric immunoassay (Cell Proliferation ELISA, BrdU colorimetric, Roche Applied Science). Cells (2500 cells/well in a 96-well plate) were incubated with 10 µM BrdU for 19 h and then fixed for 30 min. Following fixation, cells were incubated with BrDU antibody conjugated with peroxidase (anti-BrdU-POD) for 90 min at RT. Then cells were incubated with substrate (tetramethylbenzidine) for 5 min. Absorbances (A) at 370 nm and at reference wavelength (490 nm) were measured and results are expressed as A370–A490). Negative control signals from cells incubated without anti-BrDU were subtracted.

### Statistical analysis

Data expressed as mean ± standard error of the mean. “*n*” represents the number of samples used. The significance of differences was evaluated by using unpaired Student’s *t* test for two groups. *P* ≤ 0.05 was considered significant. Correlation analysis was performed using Pearson’s correlation coefficients to determine significance. In all cases, *r* ≥ 0.8 was considered evidence of positive correlation, *r* ≤ −0.8 of negative correlation. Data analyses and graphical representation were performed using GraphPad Prism 5 and RStudio.

## Results

### Differential gene expression of TRPC1-silenced Huh7 cells

**Fig. 1:**
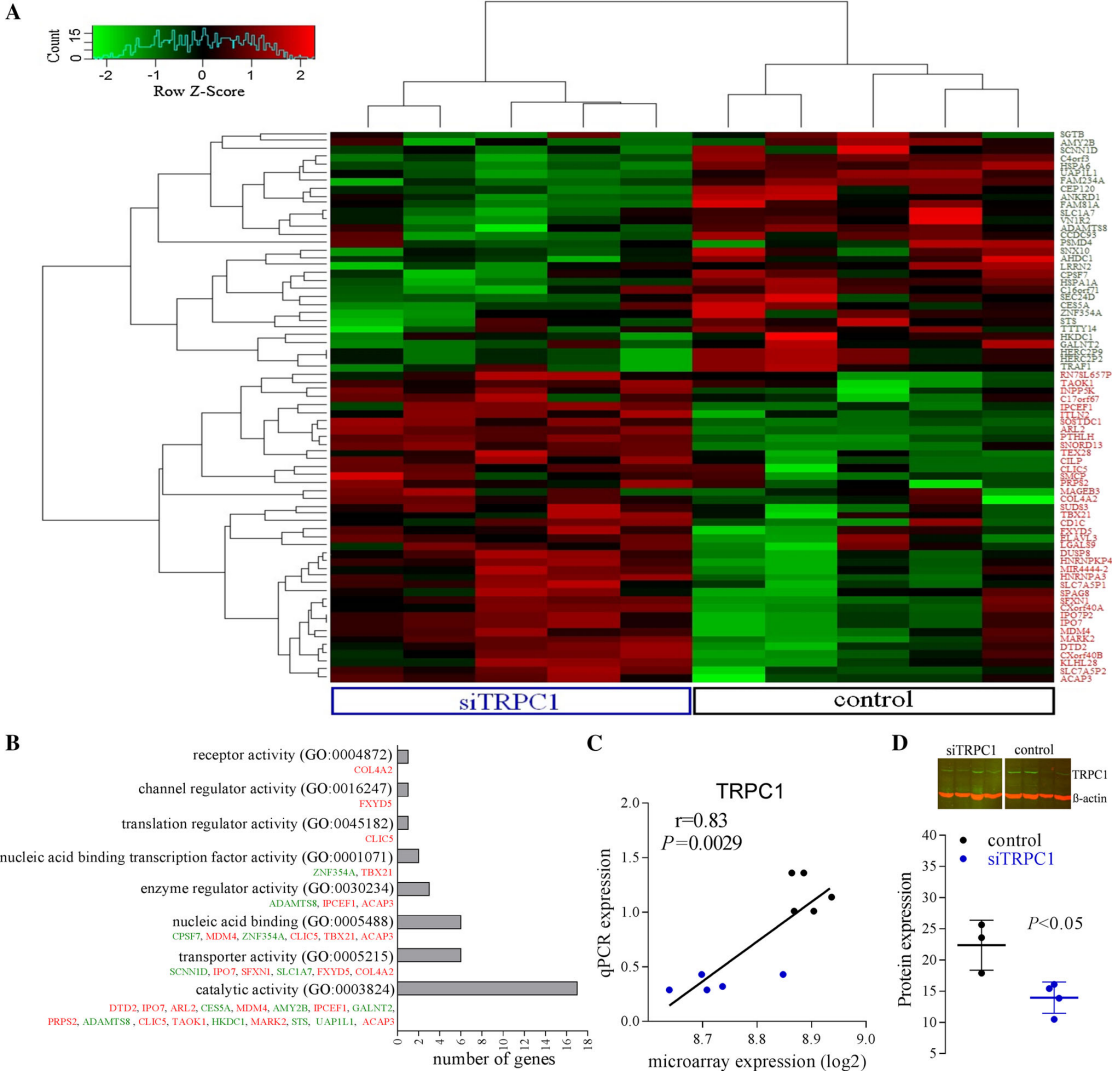
The results of differential gene expression analysis. **a** Heat map showing the clustering of samples by genes that significantly increased (*red font*) or decreased (*green font*) following *TRPC1* silencing. 40 genes were up-regulated and 31 genes down-regulated significantly in *TRPC1*-silenced (siTRPC1) cells compared to control cells. *Red* and *green* colours represent relative high and low log2 gene expression values, respectively. **b** Molecular functions of differently expressed genes. **c** Correlation of TRPC1 expression data obtained from microarray and quantitative real-time RT-PCR (siTRPC1 = *blue*, Pearson correlation, *n* = 10). **d** Effects of silencing on TRPC1 protein levels measured by Western blot following 72-h vector incubation (*n* = 4). (Color figure online)

A total of 71 genes were significantly differentially regulated (40 up; 31 down, Table 1) between *TRPC1*-silenced and control cells using Rank Product analysis (FDR, 5 %). Hierarchical clustering by differentially expressed genes is shown by heat map (Fig. 1a), where *TRPC1*-silenced and control samples distinctly cluster independently of one another. Functional analysis revealed enrichment for transporter, catalytic, and nucleic acid binding activities (Fig. 1b).

**Table 1:** The alterations in gene expression levels in *TRPC1*-silenced Huh7 cells

	Gene symbol	Description	Log2FC	FDR	*P* value
1	MARK2	MAP/microtubule affinity-regulating kinase 2	1.02	0.00050	0.000000042
2	ELAVL3	Embryonic lethal, abnormal vision, drosophila-like 3	0.94	0.00020	0.000000042
3	MDM4	Mdm4 p53 binding protein homologue (mouse)	0.89	0.00030	0.000000084
4	CXorf40A	Chromosome X open reading frame 40A	0.82	0.0014	0.00000046
5	SNORD13	Small nucleolar RNA, C/D box 13	0.81	0.0022	0.00000092
6	IPO7	Importin 7	0.82	0.0020	0.0000011
7	IPO7P2	N/A (pseudogene)	0.82	0.0020	0.0000011
8	CILP	Cartilage intermediate layer protein, nucleotide pyrophosphohydrolase	0.77	0.0030	0.0000020
9	PTHLH	Parathyroid hormone-like hormone	0.75	0.0039	0.0000029
10	ACAP3	ArfGAP with coiled-coil, ankyrin repeat and PH domains 3	0.77	0.0039	0.0000033
11	HNRNPKP4	N/A (pseudogene)	0.76	0.0042	0.0000039
12	FXYD5	FXYD domain containing ion transport regulator 5	0.77	0.0041	0.0000041
13	TAOK1	TAO kinase 1	0.72	0.0040	0.0000044
14	KLHL28	Kelch-like 28 (*drosophila*)	0.73	0.0044	0.0000051
15	HNRNPA3	Heterogeneous nuclear ribonucleoprotein A3	0.73	0.0042	0.0000053
16	SFXN1	Sideroflexin 1	0.71	0.0044	0.0000059
17	TEX28	Testis expressed 28	0.73	0.0046	0.0000066
18	ARL2	ADP-ribosylation factor-like 2	0.71	0.0045	0.0000068
19	MIR4444-2	RNA gene (MicroRNA 4444-2)	0.70	0.0066	0.000010
20	C17orf67	Chromosome 17 open reading frame 67	0.64	0.0082	0.000014
21	RN7SL657P	N/A (pseudogene)	0.65	0.013	0.000023
22	ITLN2	Intelectin 2	0.64	0.017	0.000032
23	CXorf40B	Chromosome X open reading frame 40B	0.64	0.019	0.000036
24	SPAG8	Sperm-associated antigen 8	0.60	0.020	0.000039
25	SMCP	Sperm mitochondria-associated cysteine-rich protein	0.58	0.019	0.000040
26	SLC7A5P1	Solute carrier family 7, member 5 pseudogene 1	0.64	0.019	0.000041
27	COL4A2	Collagen, type IV, alpha 2	0.64	0.021	0.000047
28	DUSP8	Dual specificity phosphatase 8	0.61	0.028	0.000066
29	PRPS2	Phosphoribosyl pyrophosphate synthetase 2	0.58	0.030	0.000072
30	CD1C	CD1c molecule	0.55	0.032	0.000080
31	TBX21	T-box 21	0.60	0.032	0.000083
32	INPP5 K	Inositol polyphosphate-5-phosphatase K	0.60	0.032	0.000085
33	SUDS3	Suppressor of defective silencing 3 homologue (*Saccharomyces cerevisiae*)	0.59	0.037	0.00010
34	IPCEF1	Interaction protein for cytohesin exchange factors 1	0.57	0.046	0.00013
35	CLIC5	Chloride intracellular channel 5	0.56	0.048	0.00014
36	SOSTDC1	Sclerostin domain containing 1	0.56	0.048	0.00014
37	DTD2	d-tyrosyl-TRNA deacylase 2 (putative)	0.58	0.047	0.00015
38	MAGEB3	Melanoma antigen family B, 3	0.47	0.047	0.00015
39	LGALS9	Lectin, galactoside-binding, soluble, 9	0.52	0.049	0.00016
40	SLC7A5P2	SLC7A5 pseudogene	0.56	0.050	0.00017
41	ADAMTS8	ADAM metallopeptidase with thrombospondin type 1 motif, 8	−0.97	0.0010	0.000000084
42	HSPA6	Heat shock 70 kDa protein 7 (HSP70B)	−0.82	0.0025	0.00000042
43	CES5A	Carboxylesterase 5A	−0.77	0.0027	0.00000067
44	HERC2P2	N/A (pseudogene)	−0.76	0.0038	0.0000014
45	HERC2P9	N/A (pseudogene)	−0.76	0.0038	0.0000014
46	HSPA1A	Heat shock 70 kDa protein 1A	−0.74	0.0045	0.0000023
47	UAP1L1	UDP-N-acteylglucosamine pyrophosphorylase 1-like 1	−0.74	0.0044	0.0000026
48	SCNN1D	Sodium channel, non-voltage-gated 1, delta	−0.73	0.0043	0.0000029
49	ANKRD1	Ankyrin repeat domain 1 (cardiac muscle)	−0.73	0.0040	0.0000030
50	AMY2B	Amylase, alpha 2B (pancreatic)	−0.64	0.012	0.0000097
51	AHDC1	AT hook, DNA binding motif, containing 1	−0.67	0.015	0.000014
52	STS	Steroid sulfatase (microsomal), isozyme S	−0.65	0.017	0.000017
53	C16orf71	Chromosome 16 open reading frame 71	−0.63	0.020	0.000022
54	ZNF354A	Zinc finger protein 354A	−0.62	0.025	0.000029
55	CCDC93	Coiled-coil domain containing 93	−0.61	0.029	0.000036
56	TRAF1	TNF receptor-associated factor 1	−0.59	0.031	0.000042
57	CEP120	Centrosomal protein 120 kDa	−0.62	0.030	0.000042
58	FAM234A	Family with sequence similarity 234 member A	−0.61	0.032	0.000049
59	GALNT2	Polypeptide *N*-Acetylgalactosaminyltransferase 2	−0.57	0.041	0.000065
60	SEC24D	SEC24 family, member D (*S. cerevisiae*)	−0.58	0.039	0.000066
61	SGTB	Small glutamine-rich tetratricopeptide repeat (TPR)-containing, beta	−0.51	0.046	0.000081
62	VN1R2	Vomeronasal 1 receptor 2	−0.61	0.044	0.000081
63	LRRN2	Leucine rich repeat neuronal 2	−0.58	0.043	0.000083
64	PSMD4	Proteasome (prosome, macropain) 26S subunit, non-ATPase, 4	−0.38	0.043	0.000086
65	FAM81A	Family with sequence similarity 81, member A	−0.58	0.045	0.000094
66	SNX10	Sorting nexin 10	−0.55	0.044	0.000095
67	TTTY14	Testis-specific transcript, Y-linked 14	−0.55	0.043	0.000097
68	HKDC1	Hexokinase domain containing 1	−0.56	0.043	0.00010
69	SLC1A7	Solute carrier family 1 (glutamate transporter), member 7	−0.59	0.044	0.00011
70	C4orf3	Chromosome 4 open reading frame 3	−0.57	0.049	0.00012
71	CPSF7	Cleavage and polyadenylation specific factor 7, 59 kDa	−0.56	0.048	0.00012

MAPK signalling pathway was observed to be significantly enriched (enrichment score = 1.02, *P* = 0.04, fold enrichment = 4.74, FDR = 27 %). TAO kinase 1 (TAOK1), dual specificity phosphatase 8 (DUSP8), HSPA6, and HSPA1A genes were clustered in this pathway. TAOK1 (log2FC = 0.72*, P* = 4.39E − 06) and DUSP8 levels (log2FC = 0.61*, P* = 6.63E − 05) up-regulated, respectively, whereas heat shock protein genes HSPA6 and HSPA1A were down-regulated in silenced cells (log2FC = −0.82, *P* = 4.18E − 07 and log2FC = −0.74, *P* = 2.26E − 06, respectively). Genes associated with Ca^2+^ entry mechanisms, for example, Ca^2+^ pumps and exchangers located on ER (SERCA) and plasma membrane (PMCA and NCX), were not differentially expressed in *TRPC1*-diminished cells.

**Fig. 2:**
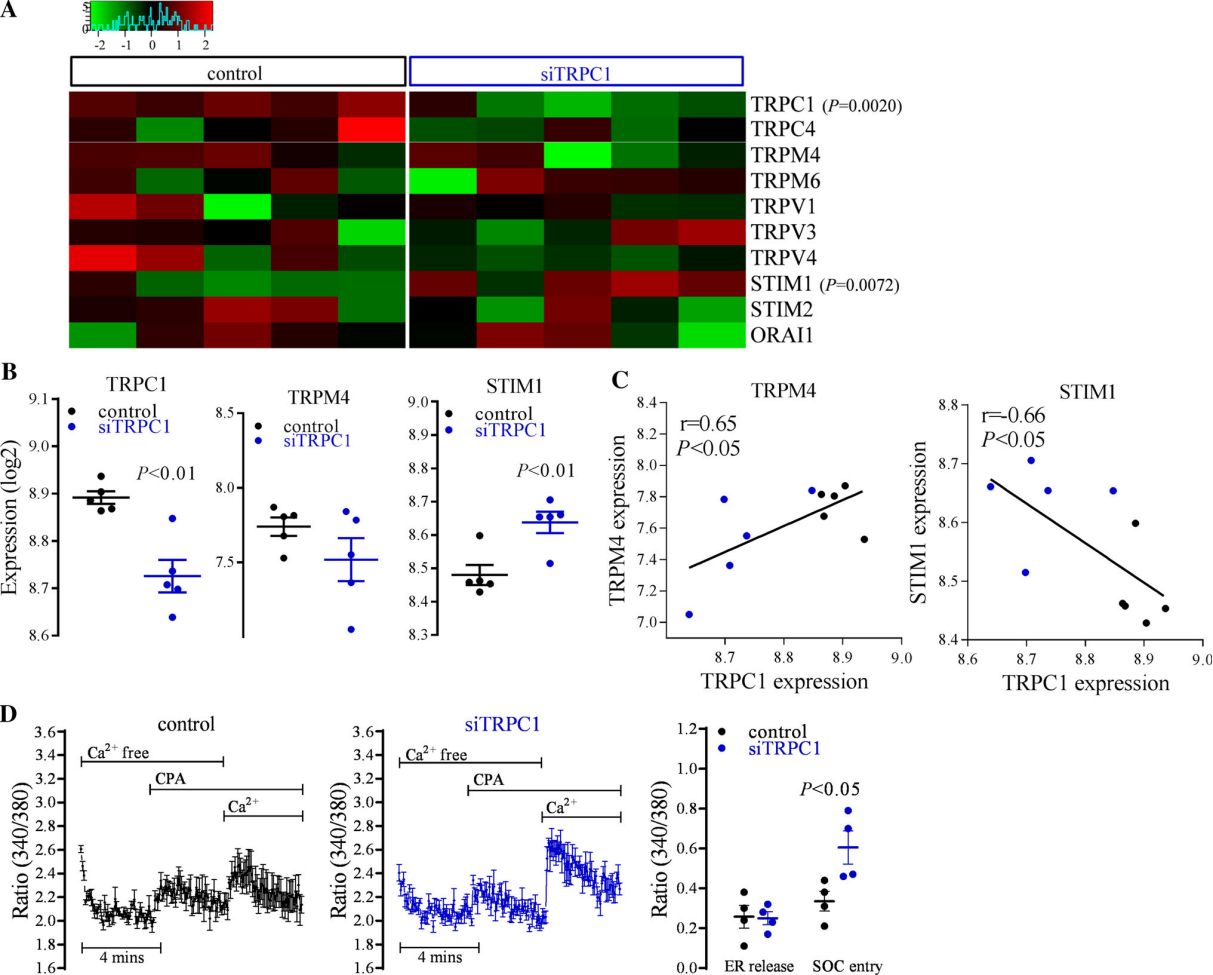
Changes in SOC entry-related genes’ expression and SOC entry following TRPC1 silencing. **a** Heat map showing the SOC entry-related gene expression (log2 values) following *TRPC1* silencing. TRP family members, STIM, and Orai1 proteins with detectable gene expression were included in the heat map. *Red* and *green* colours represent relative high and low log2 gene expression values, respectively. **b** Changes in TRPC1, TRPM4, and STIM1 expression in control and *TRPC1*-silenced (siTRPC1) cells (*n* = 5). **c** Correlation of TRPC1 expression with TRPM4 and STIM1 (siTRPC1 = *blue*, Pearson correlation, *n* = 10). **d** Cyclopiazonic acid (CPA)-induced Ca^2+^ elevations and the cumulative data of SOC entry in control and *TRPC1*-silenced cells. Changes in endoplasmic reticulum (ER) Ca^2+^ release and store-operated Ca^2+^ (SOC) entry are shown (*n* = 4). Changes in peak responses were evaluated and expressed as ratio (340/380). (Color figure online)

TRPC1 expression was significantly (*P* ≤ 0.01, *n* = 5) decreased in silenced cells (Fig. 2a, b) in both microarray and subsequent quantitative real-time RT-PCR validation experiments. TRPC1 expression was strongly correlated between PCR and microarray in 10 out of 12 samples (*r* = 0.83, *P* ≤ 0.01, *n* = 10), indicating successful *TRPC1* silencing (Fig. 1c). Samples 9 and 10 (GSE77386) were excluded in data analysis due to low correlation. TRPC1 protein levels significantly suppressed by 41 % (Fig. 1d, *P* ≤ 0.05, *n* = 4), demonstrating effective TRPC1 silencing.

### Changes in SOC entry-related genes’ expression and SOC entry following TRPC1 silencing

We further analysed expression changes in SOC entry-related genes, including all TRP family members, STIM1, STIM2, and orai1. TRPA1, TRPC3, C5, C6, C7, TRPN, TRPM1, M2, M3, M5, M7, M8, TRPML1, ML2, ML3, TRPP2, P3, P5, TRPV2, V5, V6, orai2, and orai3 failed to pass the microarray detection threshold as they were not detectable above the background levels on the microarray. The heat map illustrates detectable gene expression of TRP family members, STIM, and orai1 in control and *TRPC1*-silenced cells (Fig. 2a).

In contrast to significantly down-regulated TRPC1 levels, STIM1 levels were up-regulated significantly (*P* ≤ 0.01, *n* = 5) without changes in the orai1 levels following *TRPC1* silencing (Fig. 2b). Aside from TRPC1 and STIM1, *TRPC1* silencing did not significantly alter other SOC entry-related genes.

Significant correlations between TRPC1 expression with TRPM4 and STIM1 were observed (Fig. 2a). TRPM4 levels were positively correlated with that of TRPC1 (*r* = 0.65, *P* ≤ 0.05, *n* = 10) (Fig. 2c), although no significant changes in its expression following *TRPC1* silencing were observed (*n* = 5) (Fig. 2b). In addition, TRPC1 expression was negatively correlated with that of STIM1 (*r* = −0.66, *P* ≤ 0.05, *n* = 10) (Fig. 2c).

Real-time changes in intracellular Ca^2+^ levels were monitored after 72 h of vector transfection. In *TRPC1*-silenced cells, SOC entry induced by CPA enhanced significantly (*P* ≤ 0.05, *n* = 4) without a change in ER Ca^2+^ release (Fig. 2d).

### Changes in proliferation-related genes’ expression and Huh7 cell proliferation

**Fig. 3:**
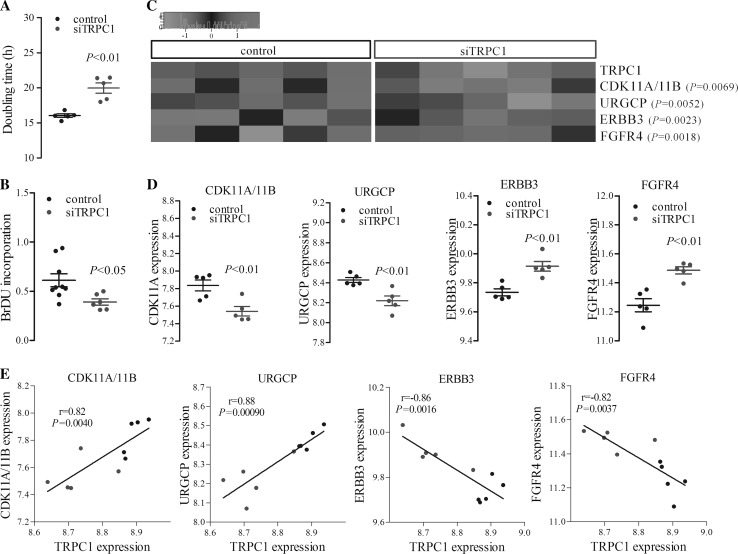
Changes in cell proliferation- and proliferation-related genes’ expression with that of TRPC1 following silencing in Huh7 cells. **a** The cumulative data of doubling time (in hour) in control and *TRPC1*-silenced (siTRPC1) cells (*n* = 5). Real-time changes in cell proliferation were evaluated and doubling time was calculated. **b** The cumulative data of cell proliferation measured by BrDU incorporation in control and *TRPC1*-silenced (siTRPC1) cells (*n* = 6). **c** Heat map showing the expression of the proliferation-related genes (CDK11A/11B, URGCP, ERBB3, and FGFR4) correlated with TRPC1 levels. *Red* and *green* colours represent relative high and low log2 gene expression values, respectively. **d** Changes in CDK11A/11B, URGCP, ERBB3, and FGFR4 expression in control and *TRPC1*-silenced cells (*n* = 5). **e** Correlation of proliferation-related genes with TRPC1 expression (siTRPC1 = *blue*, Pearson correlation, *n* = 10). (Color figure online)

Cell proliferation was monitored by using real-time cellular analysis and doubling time was calculated. Doubling time increased significantly (*P* ≤ 0.01, *n* = 5) indicating suppression of cell proliferation in *TRPC1*-silenced cells (Fig. 3a). In addition, BrDU incorporation assay showed a significant reduction of cell proliferation in *TRPC1*-silenced cells compared to control cells (*P* ≤ 0.05, *n* = 6, Fig. 3b).

A further analysis was undertaken to consider whether molecular changes following TRPC1 expression were able to explain the reduction in cell proliferation. For this purpose, hepatocellular carcinoma cell proliferation-related genes were selected, and both correlation with TRPC1 and changes in relative expression were analysed (Fig. 3c–e).

CDK11A/11B and URGCP levels were positively correlated with that of TRPC1 (*r* = 0.82 and *r* = 0.88, *P* ≤ 0.01, *n* = 10) (Fig. 3e), exhibiting a significant decrease in expression following *TRPC1* silencing (*P* ≤ 0.01, *n* = 5) (Fig. 3c, d). In addition, TRPC1 expression was negatively correlated with that of ERBB3 (*r* = −0.86, *P* ≤ 0.01, *n* = 10) and FGFR4 (*r* = −0.82, *P* ≤ 0.01, *n* = 10) (Fig. 3e). ERBB3 and FGFR4 levels were up-regulated significantly following *TRPC1* silencing (*P* ≤ 0.01, *n* = 5) (Fig. 3c, d).

## Discussion

We have previously described a negative regulatory role for TRPC1 in SOC entry, both in vascular smooth muscle and hepatocellular carcinoma cells [15, 16]. These findings are supported here, where SOC entry was significantly up-regulated following *TRPC1* silencing. In addition, we have also shown that TRPC6 levels increase in contrast to *TRPC1* down-regulation suggesting a compensatory role for TRPC6 in SOC entry in vascular smooth muscle cells [15]. However, it has also been demonstrated that TRPC6 levels remain unaltered by *TRPC1* silencing in Huh7 cells [16]. TRPC6 up-regulation was unable to be replicated in the present study due to the limitations of the microarray technology, where TRPC6 was not detectably expressed in our samples. In addition to TRPC1, TRPM4 was recently shown to be a negative regulator of SOC entry [21]. Although TRPM4 levels were positively correlated with down-regulation of *TRPC1*, TRPM4 expression was not significantly changed in our study possibly indicating that they function independently but also potentially due to tissue-specific variations in SOC entry regulatory mechanisms. In addition, the level of STIM1, another purported regulator of SOC entry and TRPC1 function [13], significantly increased suggesting an interplay between STIM1 and TRPC1 in the elevation of SOC entry. STIM1 has been shown to be critical for the activation and coupling of TRPM4 cation channel with Ca^2+^ stores in myocytes [22]. Based on this, reciprocal up-regulation of STIM1 suggests a compensatory or contributory role of STIM1 in the up-regulation of SOC entry in cancer cells. The changes in protein levels of mentioned channel components in SOC entry up-regulated hepatocellular carcinoma cells need further investigation.

Our previous observations demonstrated that *TRPC1* silencing suppresses cell proliferation without affecting cell migration in Huh7 cells suggesting a regulatory role of TRPC1 in the proliferation of hepatocellular carcinoma cells [16]. In contrast to this, Huh7 cell proliferation was shown to be related with SOC entry and TRPC6 but not with TRPC1 [5]. In the present study, the doubling time of *TRPC1*-silenced Huh7 cells was significantly longer and cell proliferation measured by BrDU incorporation was significantly lower than that of control cells, further supporting the anti-proliferative role of TRPC1.

TRPM4 has also been suggested to regulate cell proliferation, where suppression of TRPM4 decreased cervical cancer cell proliferation via β-catenin degradation [23]. It is well documented that Wnt/β-catenin signalling plays a key role in the development of hepatocellular carcinoma [24]. Here, TRPM4 levels were positively correlated with *TRPC1* down-regulation suggesting a possible inhibitory role for TRPM4 as well as TRPC1 in Huh7 cell proliferation. Silencing of STIM1 has also been shown to inhibit hepatocellular carcinoma cell proliferation by cell cycle arrest [25]. In contrast, we show that cell proliferation is suppressed in STIM1 up-regulated cells following *TRPC1* silencing. Since levels of TRPC1 and SOC entry were not determined by Wu et al., the discrepancy may result from varying levels of SOC entry and SOC entry channel composition. However, we observed alterations in TRPM4 and STIM1 levels in the context of *TRPC1* silencing, and therefore other genes and processes modulated by *TRPC1* knockdown may also participate in decreased proliferation.

*TRPC1* down-regulation along with SOC entry inhibition can result in reduced cyclin levels, G0/G1 cell cycle arrest and decreased cell growth in non-small cell lung carcinoma cell lines [26]. *TRPC1* knockdown has previously been associated with reduced amplitude of SOC entry and arrest of the endothelial progenitor cell cycle in G1 phase [27]. In addition, SOC entry was regulated at cell cycle checkpoints and shown to be at control levels during G1, up-regulated during S phase whilst suppressed during mitosis in mast cells [28]. Since we observed SOC entry up-regulation in a mixed population of cells, further cell cycle analyses with *TRPC1*-silenced cells separated into different cell cycle phases are needed to relate SOC entry levels with the cell cycle. The present analysis of cell cycle- and proliferation-associated genes revealed CDK11A/11B (CDC2L2/L1) down-regulation along with *TRPC1* silencing, suggesting cell cycle arrest. In a recent study, following CDK11 down-regulation, breast cancer cells were shown to arrest in G1 phase suggesting a critical role for CDK11 in proliferation [29]. In our study, mRNA levels of a further cell cycle-associated gene, up-regulator of cell proliferation (URGCP/URG4), decreased in parallel with decreasing cell proliferation suggesting the contribution of URGCP in Huh7 cell proliferation. URGCP/URG4 is associated with poor prognosis in hepatocellular carcinoma and its overexpression increased cellular entry into the G1/S transitional phase in hepatocellular carcinoma cells [30]. Furthermore, down-regulation of URG4 was shown to suppress cyclin D1 expression and cell proliferation in HepG2 hepatocellular carcinoma cells [31], further supporting our results.

In addition to negative regulators of cell proliferation, a number of pro-survival genes were up-regulated following *TRPC1* silencing, suggesting a compensatory gene regulation. Growth factor receptor ERBB3 and transforming growth factor alpha (TGF-α) were recently shown to act as compensatory survival factors that increased after c-Met inhibition in hepatocellular carcinoma cells [32]. Up-regulation of ERBB3 in our study may play a similar compensatory role in the suppression of *TRPC1*-silenced cell proliferation. Furthermore, up-regulation of fibroblast growth factor receptor 4 (FGFR4) levels, a target for new small molecule inhibitors for the treatment of hepatocellular carcinoma [33], may also compensate the inhibition of proliferation.

Based on differential expression analysis, we observed significant alterations in genes involved in MAPK signalling which has been associated with hepatocarcinogenesis [34]. The levels of TAO kinase 1, which activates p38 MAPK cascades and is a potential therapeutic target for cancers with defective β-catenin signalling such as hepatocellular carcinoma [24], were significantly up-regulated. On the other hand, levels of DUSP8, a negative feedback regulator of MAPKs such as p38, were significantly increased suggesting deregulation of MAPK signalling members in the presence of *TRPC1*-down-regulation.

**Fig. 4:**
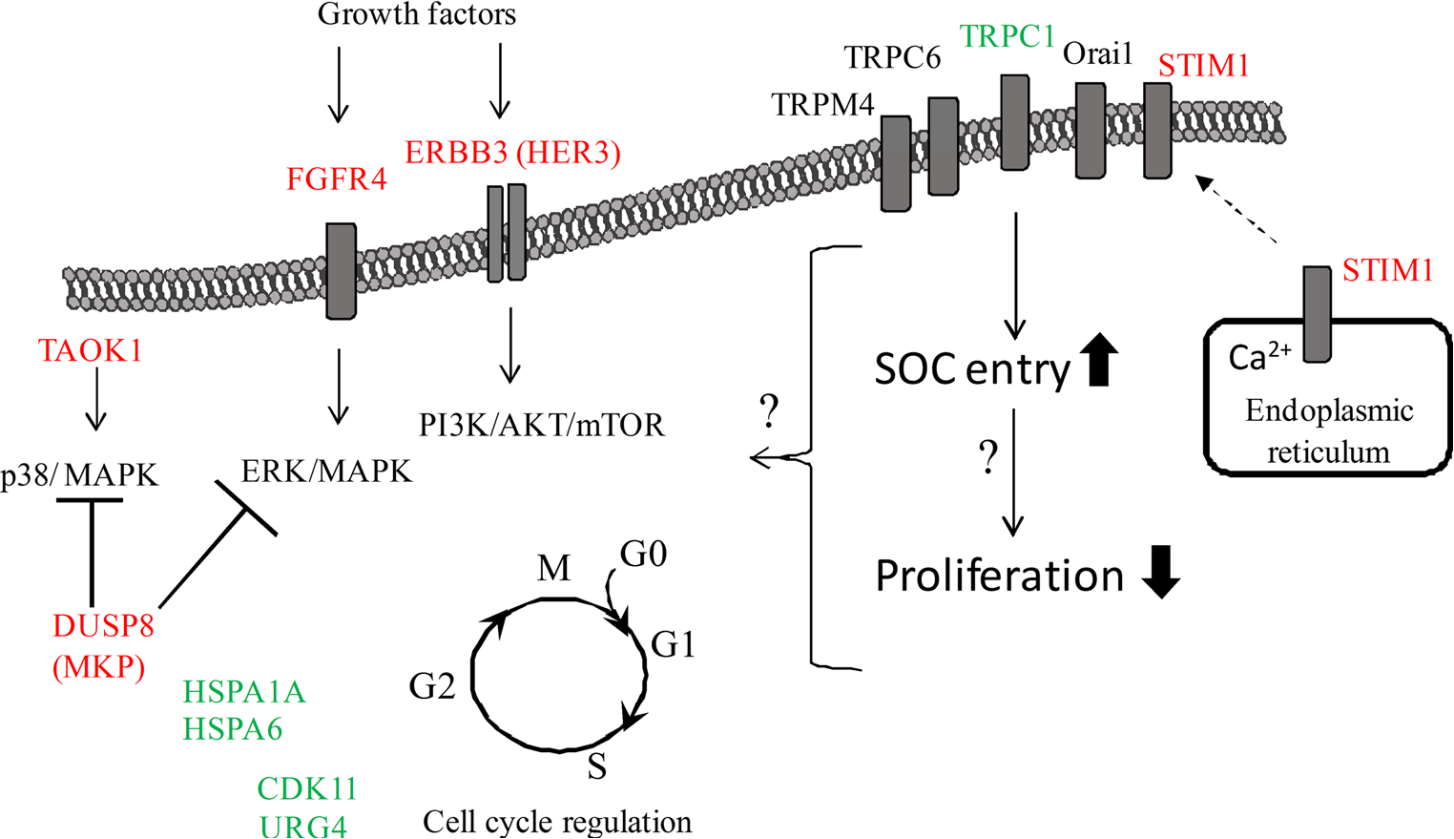
A working model showing the possible consequences of TRPC1-down-regulation in Huh7 hepatocellular carcinoma cells. Functional analysis indicated that SOC entry and cell doubling time were enhanced significantly following *TRPC1* silencing. MAPK signalling genes, DUSP8, TAOK1, HSPA6, and HSPA1A, were shown to be differentially expressed. Proliferation-related genes that were up- and down-regulated in microarray analysis are shown in *red* and *green* text, respectively. The genes that were not differentially expressed are in *black*. *Light arrows* indicate the activation and *capped lines* indicate the inhibition of the targets. *CDK11* cyclin-dependent protein kinase 11, *DUSP8* dual specificity phosphatase 8, *ErbB3 (HER3)* human epidermal growth factor receptor 3, *FGFR4* fibroblast growth factor receptor 4, *HSPA1A* heat shock protein 1A, *HSPA6* heat shock protein 6, *MAPK* mitogen-activated protein kinase, *Orai1* calcium release-activated calcium channel protein 1, *PI3K/Akt/mTOR* phosphatidylinositide 3-kinase/Akt/mammalian target of rapamycin, *STIM1* stromal interaction protein 1, *SOC* store-operated Ca^2+^, *TAOK1* thousand and one amino acid kinase 1, *TRPC* transient receptor potential channel canonical, *TRPM*, transient receptor potential channel melastatin, *URG4 (URGCP)* up-regulator of gene expression 4. (Color figure online)

A putative working model of the genes and pathways with observed altered expression in *TRPC1*-silenced Huh7 cells is given in Fig. 4. A limitation of our study is the lack of protein-level data that would reinforce our findings. In addition, the validation of the functional roles of the proposed genes in the TRPC1-mediated up-regulation of SOC entry and suppression of cell proliferation requires further investigation.

## Conclusion

In conclusion, our results show for the first time, the transcriptome-wide changes in *TRPC1*-silenced Huh7 cells. Reciprocal alterations in the levels of TRPC1 and STIM1 have been observed, suggesting their interaction in regulating SOC entry in Huh7 hepatocellular carcinoma cells. Decreased TRPC1 may be compensated by increased expression of STIM1 that possibly takes part in the up-regulation of SOC entry in *TRPC1*-silenced hepatocellular carcinoma cells. Our results further indicate the possible roles of STIM1 and TRPM4, in addition to TRPC1, in Huh7 cell proliferation by regulation of SOC entry. Alterations in MAPK signalling genes may be involved in diminished cell proliferation in *TRPC1*-silenced Huh7 cells. Moreover, alterations in cell cycle regulation genes in *TRPC1*-diminished cells suggest cell cycle arrest as well as up-regulation of possible compensatory genes to maintain cell growth. The data expressed in the current study are based on microarray expression and may need to be confirmed by further studies. The functional link between the proposed cellular processes and TRPC1 awaits further investigation.
